# Deficient Sarcolemma Repair in ALS: A Novel Mechanism with Therapeutic Potential

**DOI:** 10.3390/cells11203263

**Published:** 2022-10-17

**Authors:** Ang Li, Jianxun Yi, Xuejun Li, Li Dong, Lyle W. Ostrow, Jianjie Ma, Jingsong Zhou

**Affiliations:** 1 Department of Kinesiology, College of Nursing and Health Innovation, University of Texas at Arlington, Arlington, TX 76019, USA; 2 Department of Neurology, Lewis Katz School of Medicine at Temple University, Philadelphia, PA 19122, USA; 3 Department of Surgery, University of Virginia, Charlottesville, VA 22903, USA

**Keywords:** sarcolemma permeability, MG53, membrane repair, ROS, ALS

## Abstract

The plasma membrane (sarcolemma) of skeletal muscle myofibers is susceptible to injury caused by physical and chemical stresses during normal daily movement and/or under disease conditions. These acute plasma membrane disruptions are normally compensated by an intrinsic membrane resealing process involving interactions of multiple intracellular proteins including dysferlin, annexin, caveolin, and Mitsugumin 53 (MG53)/TRIM72. There is new evidence for compromised muscle sarcolemma repair mechanisms in Amyotrophic Lateral Sclerosis (ALS). Mitochondrial dysfunction in proximity to neuromuscular junctions (NMJs) increases oxidative stress, triggering MG53 aggregation and loss of its function. Compromised membrane repair further worsens sarcolemma fragility and amplifies oxidative stress in a vicious cycle. This article is to review existing literature supporting the concept that ALS is a disease of oxidative-stress induced disruption of muscle membrane repair that compromise the integrity of the NMJs and hence augmenting muscle membrane repair mechanisms could represent a viable therapeutic strategy for ALS.

## 1. Introduction

ALS is a fatal neuromuscular disease characterized by progressive motor neuron loss and muscle wasting. Riluzole (Rilutek) and Edaravone (Radicava), the two FDA approved treatments for ALS, demonstrate only limited efficacy to slow disease progression [1]. In most cases, the disease may be the product of multiple inter-related factors, with many efforts focused on identifying distinct patient subsets using various integrated-omics approaches [2]. While diverse cell types, biological mechanisms, and genetic factors are implicated in sporadic and familial ALS pathogenesis, there are also commonly shared pathological and clinical features [3]. Progressive respiratory muscle weakness is a main cause of morbidity and eventually death in all forms of ALS [4,5,6].

Motor neurons communicate with individual muscle fibers at neuromuscular junctions (NMJs), and retrograde signals are also conducted from muscle back to nerve [7]. While ALS is classically considered a “dying-forward” process starting in motor neurons, accumulating evidence [8,9,10,11,12,13,14,15,16,17] also implicates early muscle cell dysfunction in ALS pathophysiology. The degree to which a “dying-back” process [18,19,20] starting distally at NMJs or “dying-forward” from the CNS contributes to ALS progression remains unsettled and may vary in different patient subsets. Regardless of the direction of communication and relative contribution of different cell types to ALS progression (e.g., glia, neurons, myofibers), NMJ loss and the resulting skeletal muscle denervation is a critical early pathogenic event in both patients and animal models [21,22,23,24]. Because NMJ is the critical site involving bidirectional crosstalk between myofibers and the motor neurons [13,16,18,19,20,25,26,27], sustaining NJM integrity may slow disease progression. The molecular mechanisms accounting for NMJ loss and myofiber degeneration in ALS have attracted more attention in recent years, as strategies to slow these processes and/or enhance intrinsic muscle repair/regeneration could have broad therapeutic significance across different ALS subtypes [28].

Owing to the discovery of multiple genes that constitute the key components of cell membrane repair machinery, including dysferlin, annexin, caveolin, and MG53/TRIM72, etc., multiple review articles have been made that address the membrane repair defects linking to muscular dystrophy and heart failure, etc. [29,30,31,32,33]. However, currently there is none on the topic of membrane repair defects in ALS. This article is to review existing literature supporting the concept that ALS is a disease of oxidative-stress induced disruption of muscle membrane repair that compromise the integrity of the NMJ and hence augmenting membrane repair mechanisms could represent a viable therapeutic strategy for ALS.

## 2. Mitochondrial Dysfunction and Oxidative Stress in ALS Skeletal Muscle

Skeletal muscle is responsible for voluntary movements of the entire body, thus comprises one of the largest and most metabolically active tissues [34]. Given these energetic demands, it is not surprising that mitochondria account for 4–15% of myofiber volume [35]. Mitochondrial dysfunction is regarded as a major contributor to ALS pathology [36,37]. Both familial and sporadic ALS patients show striking mitochondrial defects and abnormalities of oxidative stress in motor neurons and skeletal muscle [38,39,40,41,42,43]. While mitochondrial dysfunction is a major source of excessive production of reactive oxygen species (ROS) leading oxidative stress, mitochondria themselves are a known target of oxidative stress due to the high sensitivity of their membrane and mtDNA to ROS. It is believed that mitochondrial dysfunction and oxidative stress play key role in neuromuscular degeneration in ALS [44,45,46]. 

Mouse models expressing human ALS mutations (e.g., SOD1^G93A^ (G93A)) recapitulate many features of human disease [47], and have been widely used to investigate pathogenic mechanisms and preclinical therapies for ALS [37,48,49,50,51]. The G93A mice demonstrate early mitochondrial dysfunction and enhanced reactive oxygen species (ROS) production in skeletal muscle [14,15,16,26,52]. Markedly elevated skeletal muscle ROS production is also an early abnormality in muscles from other ALS mouse models such as mice knockout for TDP-43 and VAPB [4,53,54,55]. The ROS accumulation in G93A skeletal muscle has been revealed by proteomics, biochemical and enzymatic assays [52,53,56]. Our prior studies demonstrated increased cytosolic and mitochondrial ROS levels in G93A myofibers beginning prior to symptom onset [14]. In this study, we generated a double transgenic mouse model (G93A/mt-cpYFP) to visualize dynamic ROS-related “mitoflash” events in G93A myofibers [14,26]. “Mitoflash” events are spontaneous fluorescent transients of the mitochondrial targeted, circularly permuted yellow fluorescent protein (mt-cpYFP), and has been associated with mitochondrial ROS production [57]. Widespread mitoflash activities has been revealed in myofibers derived from G93A/mt-cpYFP mice prior to symptom onset, which was not seen in wild type (WT) mt-cpYFP myofibers. The increased mitoflash activity is associated with increased opening of mitochondrial transition pores (mPTP) [26,57,58,59,60]. mPTP opening can promote mitochondrial ROS production [61,62]. We also detected an elevated level of the mitochondrial protein cyclophilin D (CypD) in G93A skeletal muscle [14], which is accompanied by abnormalities in mitochondrial structure and function [11,13,16]. CypD is a known activator of mPTP opening [63,64,65,66,67] and CypD-related mPTP opening is tightly associated with mitoflash activities [68,69,70]. Enhanced CypD has been associated with more frequent and widespread mitoflash activities in myofibers [58,71,72]. Thus, CypD-mediated mPTP opening may be one of the underlying mechanisms to enhanced ROS production in ALS muscle. However, it is worth to note that increased CypD level may not be a solo cause of excessive ROS generation in ALS skeletal muscle. Other factors, such as mitochondrial Ca^2+^ overload, oxidizing agents, thiol oxidation, HSP90., etc., also promote high-conductance opening of mPTP in pathological conditions including ALS [73]. 

## 3. ALS Mice Exhibit Sarcolemma Fragility and Mitochondrial Dysfunction in Proximity to NMJs Prior to Symptom Onset

Intraperitoneal injection of Evans Blue dye (EB) is widely used as an in vivo marker of abnormal myofiber permeability from sarcolemma damage [74,75,76,77]. We observed consistently elevated intracellular EB penetration in the diaphragm and tibialis anterior muscles of G93A mice after downhill running [78]. Even without the exercise challenge, a significant portion of diaphragm myofibers in 2-month-old G93A mice (pre-symptomatic) showed EB penetration. The phenomenon became more prominent when the G93A mice reached 4-month of age (i.e., after symptom onset). In contrast, EB penetration was not seen in the diaphragm of WT controls, with or without running, at 2- or 4-months of age [78]. These observations indicate that the increase of sarcolemma vulnerability occurs early in the course of the disease in ALS mice. 

The enhanced sarcolemma fragility is most striking near NMJs. After downhill running, isolated flexor digitorum brevis (FDB) myofibers from 2-month-old G93A mice exhibited intracellular accumulation of FM 1-43, a cell membrane impermeable dye often used for ex vivo evaluation of sarcolemma integrity [30,77,79], forming a gradient centered around the NMJ. This phenomenon was not observed in myofibers derived from WT mice [78]. These data suggest that NMJs are focally more susceptible to injury than other regions of the sarcolemma, and exercise-induced NMJ injury is exacerbated in G93A mice before ALS symptom onset. Remarkably, mitochondrial lesion appears first at the region harboring NMJ in the G93A muscle with a myofiber segment showing mitochondrial inner membrane depolarization prior to ALS symptom onset, and this myofiber segment at the NMJ with depolarized mitochondria exhibits uncontrolled hyperactive Ca^2+^ activity, which should never occur in normal WT myofibers [13,16]. This early mitochondrial lesion in proximity to NMJ may be a cause of sarcolemma damage initiated at NMJ. 

Nicotinic acetylcholine receptors (nAchR) are abundant in the postsynaptic membrane of NMJ. There is evidence of an elevated Ca^2+^ permeability of nAchR in adult mammalian muscle [80], and increased nAchR expression was found in G93A muscle [81]. It can be expected that following repetitive stimulations, mitochondria near NMJ could face elevated local intracellular [Ca^2+^], leading to mitochondrial Ca^2+^ overload at NMJ [15]. Mitochondrial Ca^2+^ overload also triggers mPTP opening, which causes mitochondrial depolarization and promotes ROS generation [62]. The depolarized mitochondria have reduced capacity to take up Ca^2+^, thus, an increased cytosolic Ca^2+^ release events were observed at region near NMJ of G93A myofibers [13,16]. This abnormal Ca^2+^ release could further stress neighboring normal mitochondria with Ca^2+^ overload, causing mPTP opening, mitochondrial depolarization and excessive ROS generation in a vicious cycle started from NMJ. As the cell membrane is a major target of direct ROS attack [46,82], it is possible that early mitochondrial lesion with excessive ROS near NMJ initiates the membrane leakage observed at NMJ in G93A myofibers. Furthermore, those lesions could propagate and affect the entire myofiber during ALS disease progression, although the underlying molecular natures need to be further explored. In addition to damaging the lipid membrane of sarcolemma, elevated ROS level could also affect the normal membrane repair mechanism, which will be further discussed below. Indeed, disorganized T-tubule networks were observed near the NMJ in the myofibers of G93A mice before ALS symptom onset, becoming more striking at later stages of disease. The degree of T-tubule disorganization correlated with the abnormal mitochondrial depolarization at NMJs [78]. As skeletal muscle mitochondrial dysfunction and enhanced oxidative stress are described in different genetic and sporadic ALS subtypes, the resulting sarcolemma fragility may similarly be a common phenomenon. 

## 4. MG53-Mediated Membrane Repair Is Compromised in ALS

The stress to myofiber integrity resulting from repeated muscle contraction-relaxation (such as in the diaphragm muscle during respiration) is offset by intrinsic sarcolemma repair mechanisms. The dynamic balance between potentially injurious stress and membrane repair can be shifted towards myofiber damage in situations where a disease state amplifies the stress beyond the intrinsic capacity for repair, and/or disrupts the repair mechanisms themselves. MG53 is a member of the tri-partite motif (TRIM) E3-ligase family protein (encoded by *TRIM72*) [83], which is highly expressed in skeletal muscle [84]. Upon acute plasma membrane injury, MG53 acts as a sensor to oxidized intracellular microenvironments, and then facilitates trafficking of intracellular vesicles to form membrane repair patches at focal sites of injured sarcolemma. MG53 is the molecule that first arrives at the injury site (within 2 s following sarcolemma rupture) and plays an essential role in maintaining sarcolemma integrity [77,85,86,87]. Genetic ablation of MG53 results in defective membrane repair and diminished tissue regenerative capacity [77,85,88,89]. Conversely, transgenic mice with sustained elevation of MG53 in the bloodstream (tPA-MG53) live a long, healthy life span with enhanced tissue-regenerative capacity following injury [90].

We have demonstrated that MG53 forms membrane patches on the sarcolemma specifically in proximity to NMJs to preserve NMJ integrity under normal physiological condition [78]. Impaired MG53-related membrane repair function could play an essential role in the progressive degeneration of NMJs in ALS. As discussed above, mitochondrial dysfunction is a major cause of excessive ROS production in ALS muscle [14,38,39,40,41,42,43,52]. Our studies suggest that the prominent and persistently enhanced oxidative stress in ALS muscle limits the movement of intracellular MG53 vesicles, causing abnormal MG53 protein aggregation. The intracellular aggregation of MG53 protein could compromise the MG53-related sarcolemma repair mechanism. We observed pathologic MG53 aggregation in all examined muscle types of the G93A mice including fast and slow twitch muscles, and importantly also in postmortem human diaphragm and psoas muscles samples from both sporadic and familial ALS patients (harboring different ALS mutations), but not in controls. [78]. Therefore, compromised MG53-mediated membrane repair function appears to be a common feature of different familial and sporadic ALS subtypes. 

The effects of exercise training on ALS progression remains controversial [91,92]. Several published cohort studies suggest an association between intense physical activities and increased risk of ALS [93,94,95,96]. Furthermore, the effects of exercise training on ALS progression remains controversial, with some studies suggesting modest benefits from mild or moderate exercise while others, especially of more strenuous physical exertion, may be detrimental [91,92]. A trial of diaphragm muscle pacing reduced survival in ALS patients with respiratory insufficiency [97,98,99,100,101]. Similarly, in our ALS mouse studies, even modest exercise worsened diaphragm damage [78].

## 5. Therapeutic Potential of Exogenously Administered MG53 in ALS

We evaluated the potential efficacy of recombinant human MG53 (rhMG53) using the ALS G93A mouse model [78]. Adding rhMG53 to the extracellular solution significantly attenuated intracellular FM 1-43 accumulation in G93A myofibers in vitro. We treated 3-month-old G93A mice (after symptom onset) with intravenous rhMG53 (2 mg/kg body weight) once daily for 2 weeks. The rhMG53 injected mice exhibited less denervated NMJs in the diaphragm and more surviving motor neuron cell bodies in the spinal cord anterior horns compared with the saline injected control groups [78]. We also produced a PEGylated rhMG53 (PEG-rhMG53) protein with increased half-life of rhMG53 in circulation [102,103]. PEG-rhMG53 was similarly administered to three-month-old G93A mice (2 mg/kg body weight, every other day for one month). The life span of the PEG-rhMG53 injected mice was significantly prolonged for 13 days on average compared with the saline injected group, and the benefits were observed in both male and female mice. The PEG-rhMG53 treatment also slowed weight loss [78].

As MG53 is present at low levels in circulation under normal physiologic conditions in human and rodents [104,105,106,107], administration of exogenous rhMG53 is not likely to produce neutralizing antibodies as peripheral tolerance to this protein has already occurred. Studies in multiple mouse models reported no observable toxic effects with long-term administration of rhMG53 [104,108]. rhMG53 protein has been found to protect various cell types against membrane disruption, and ameliorate the pathology associated with muscular dystrophy [104], acute lung injury [109], myocardial infarction [110], acute kidney injury [111] and ischemic brain damage [112] in rodent and large animal models of these diseases. 

MG53 protein is released from skeletal muscle as a myokine [33]. We previously demonstrated elevated serum levels of endogenous MG53 in a mouse model of muscular dystrophy (*mdx*) compared to WT mice [104]. Similarly, serum MG53 was markedly elevated in the 2-month-old G93A mice after downhill running—likely a reflection of enhanced skeletal muscle membrane injury [78]. Interestingly, serum MG53 levels in later stage ALS mice was reduced to levels lower than in WT mice. This reduced serum MG53 level could be due to muscle atrophy in later stage of disease, perhaps combined with diminished MG53 secretion due to the pathological aggregation we observed with disease progression in the ALS mice. 

## 6. Conclusions and Future Perspectives 

**Figure 1:**
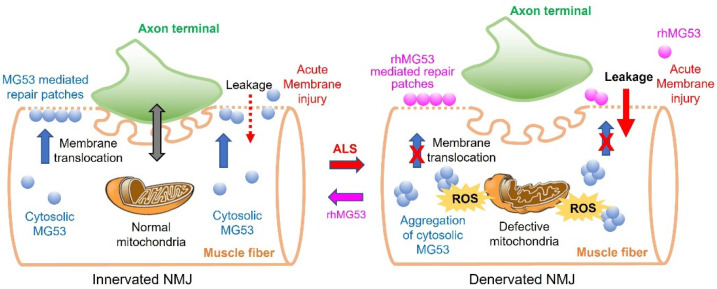
Proposed mechanisms of membrane repair defects at NMJ in ALS and the restoration by exogenous rhMG53. NMJ is the critical site of neuromuscular interactions. During ALS progression, abnormalities in mitochondrial respiratory activities at NMJ elevates ROS production, leading to ectopic aggregation of cytosolic MG53, undermining its membrane repair function, which exacerbates sarcolemma disruption leading to accumulation of extracellular content in the cytosol. Exogenously applied rhMG53 could be recruited to the sites of membrane injury, forming sealing patches to alleviate the cell membrane leakage.

Our early studies using the G93A mouse model established a role for mitochondria Ca^2+^ signaling and ROS in mediating the crosstalk between muscle and neurons at the NMJ during ALS progression [13,14,15,16,26]. As illustrated in Figure 1, during ALS progression, mitochondrial dysfunction in muscle myofibers leads to excessive ROS production, which promotes ectopic aggregation of cytosolic MG53 and loss of function. This worsens sarcolemma disruption, resulting in a vicious cycle of worsening oxidative stress, muscle membrane damage, and NMJ degeneration. Treating ALS mice with exogenous rhMG53 can enhance the formation of membrane sealing patches at the damaged sarcolemma, accounting for the reduced membrane leaking we observed in vitro, preservation of diaphragm muscle NMJs and motor neuron cell bodies in the spinal cord in vivo, as well as prolonging life and slowing weight loss. 

NMJs in mice and humans have different anatomical structures: the clefts of the NMJ are deeper in humans compared to mice suggesting a need for amplifying the signal in humans that is greater than in mice. Considering the difference in synaptic transmission, preservation of NMJ integrity could even be more crucial in human. Presumably, rhMG53 can have therapeutic benefits to preserve NMJ integrity in ALS patients.

Our studies in mice with a pathogenic SOD1 mutation and human autopsy muscle samples from decedents who harbored C9orf72 mutations, and “sporadic” ALS (with no known pathogenic mutation)—all showed the same abnormal MG53 aggregation, which was not seen in control mouse or human muscles. This suggests that abnormalities of MG53 function may be seen across different ALS subtypes, and thus treating with exogenous recombinant MG53 could have broad therapeutic applicability to ALS patients. Due to the heterogeneity in etiology, clinical and pathology, the treatment of ALS patients may be best achieved by a multidisciplinary approach with targeting different potential pathological mechanisms. Intriguingly, combining rhMG53 treatment with exercise and/or diaphragm pacing may protect from the worsening of disease seen in prior exercise/pacing trials, while still allowing for the beneficial effects that exercise normally has on muscle physiology and strengthening. 

MG53 is present at low levels in blood circulation under normal physiologic conditions in both rodents and humans [104,105,106,107]. Higher circulating levels of MG53 were observed in the G93A mice compared to WT littermates, and correlated with serum CK measurements [78]. If similar elevations of circulating endogenous MG53 are seen in ALS patients, it could be useful as a “prognostic biomarker” to quantify the degree of myofiber degeneration at early disease stages. Furthermore, muscle biopsy is a standardized diagnostic clinical procedure performed at clinical and academic centers around the world. While not generally part of the clinical workup for ALS, biopsies have been used in several ALS clinical trials to look for differences pre- and post-treatment, [https://www.clinicaltrials.gov/ct2/show/NCT04632225 (accessed on 6 September 2022)]. Serum levels of endogenous MG53 and/or muscle biopsy pathology could similarly be promising pharmacodynamic measures to demonstrate therapeutic efficacy for ALS intended to preserve myofiber integrity.

Although MG53 was the first molecules investigated in ALS for its role in sarcolemma repair, there are other membrane repair proteins [29] with undetermined roles in skeletal muscle degeneration in ALS. Future studies should be encouraged to further investigate whether and how those membrane repair proteins are involved in skeletal muscle wasting in ALS. This type of study should provide additional potential therapeutic targets for ALS. We wish this review article could attract more attention of the ALS community and promote research efforts to further explore the possibility of considering preservation of skeletal muscle membrane integrity as a potential therapy for ALS.
